# Teaching the Careless

**Published:** 1920-05-22

**Authors:** 


### Teaching the Careless.

Health authorities here might with advantage emulate the wisdom of the municipal council of Johannesburg, which has drawn up the subjoined new by-law in order to deal with parents who allow their infected children to run loose:—

No person who is suffering from smallpox, diphtheria, membranous croup or the disease known as scarlatina or scarlet fever, and who is under treatment elsewhere than in a recognised fever hospital, shall permit any person, not being a member of his own household or his medical or nursing or other necessary attendant, to visit or come into contact with him during the recognised period of infectivity of such disease without the written permission of the Medical Officer of Health; and no person being in charge of any child or other individual so infected and similarly under treatment shall permit such infected child or other individual to be so visited, or to be in contact with non-infected persons, without such written permission.

